# Almost a Near Miss: Hairy Cell Leukemia

**DOI:** 10.7759/cureus.33949

**Published:** 2023-01-18

**Authors:** Jasmita Parkash, Varinder Bansro, Gurdeep S Chhabra, Zainab Mujahid

**Affiliations:** 1 Internal Medicine, Dr. Rajendra Prasad Government Medical College, Himachal Pradesh, IND; 2 Internal Medicine, University of Maryland Capital Region Health, Largo, USA; 3 Hematology and Medical Oncology, University of Maryland Capital Region Health, Largo, USA

**Keywords:** massive splenomegaly, purine analogues, menorrhagia, severe iron deficiency anemia, thrombocytopenia, hairy cell leukemia

## Abstract

Hairy cell leukemia (HCL) is an infrequently encountered chronic B-lymphocyte hematological malignancy, which is found to be more prevalent in males. HCL can present with a myriad of nonspecific symptoms involving the reticuloendothelial system. Usually, patients are diagnosed after an incidental finding of pancytopenia. In the majority of cases, HCL follows an indolent course, and many patients remain asymptomatic. Treatment with nucleoside analogs is the first line of treatment and is indicated for patients with severe anemia, thrombocytopenia, neutropenia, or severe systemic symptoms. Here, we report an atypical case of a 41-year-old Hispanic female who presented with menorrhagia and iron deficiency anemia. She was diagnosed with HCL after a bone marrow biopsy demonstrated the characteristic “hairy projections."

## Introduction

Hairy cell leukemia (HCL) is an uncommon chronic clonal B-cell malignancy [1]. It accounts for 2% of all lymphoid leukemia cases [2]. Males are more affected than females with male to female ratio ranging from 4:1 to 5:1 [2]. The median age of diagnosis is 50-55 years, and it is three times more common in Caucasians as compared to African Americans. It commonly presents as pancytopenia, infections, and/or splenomegaly (due to the accumulation of neoplastic cells). Investigations usually reveal mononuclear cells with a “hairy” or “frilled” appearance on peripheral blood smear or bone marrow biopsy. Diagnosis is confirmed by a bone marrow aspiration and core biopsy with immunophenotyping and flow cytometry [3]. The majority of patients remain asymptomatic [2,3]. The rare ones that are symptomatic have complications such as anemia, bleeding, and infections [2,3]. Treatment is not curative, and it focuses on symptomatic care and prolonging lifespan [2,3]. Therapy is initiated when a patient has one or more of the following: significant cytopenias, symptomatic splenomegaly or adenopathy, and constitutional symptoms [2,3]. First-line treatment is a purine-analog-based therapy [3]. Active infections should be treated with antimicrobials prior to initiating purine analog therapy [3]. Here, we describe the following case of a patient with undiagnosed HCL who presented with complaints of menorrhagia and iron deficiency anemia.

## Case presentation

**Figure 1:**
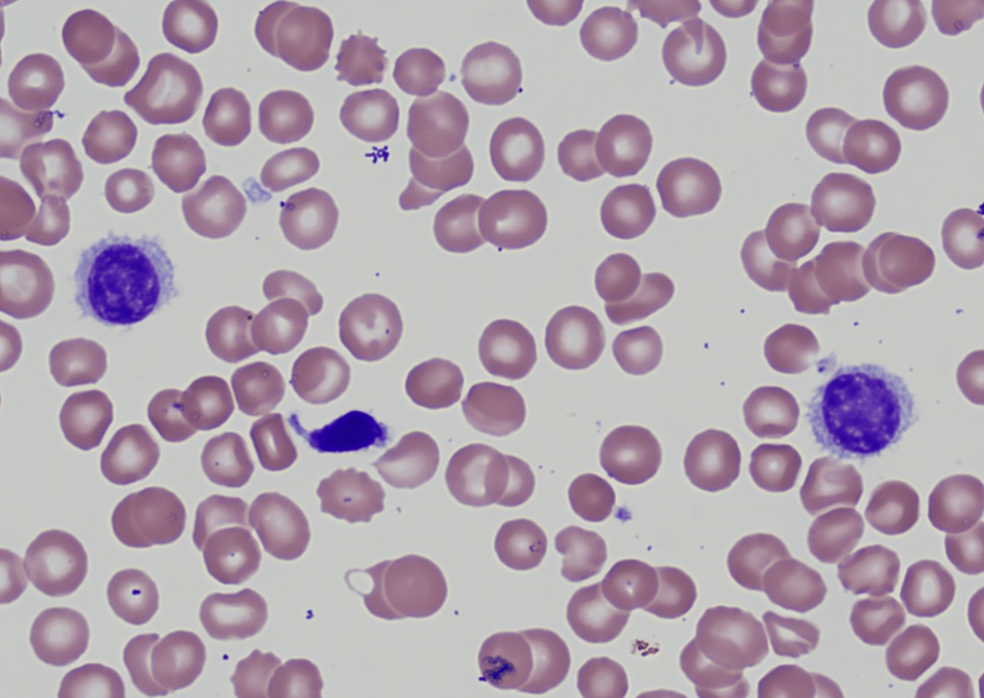
Peripheral blood smear showing characteristic hairy cell along with marked thrombocytopenia and hypochromic anemia with nucleated RBCs and anisocytosis RBCs: Red blood cells.

A 41-year-old Hispanic female with a past medical history of iron deficiency anemia and menorrhagia reported to the emergency department of a tertiary care hospital with a chief complaint of fatigue on exertion. She reported abnormal menstrual cycles with heavier bleeding recently. On examination, she was tachycardic with a pulse rate of 110 beats per minute and a blood pressure of 100/60 mmHg. Abdominal examination revealed moderate splenomegaly. Lab work performed showed hemoglobin of 2.6 g/dL, hematocrit of 9.9%, and severe thrombocytopenia with a platelet count of 28,000/µL, and the white blood count was within normal limits. She received a total of four units of packed red blood cells along with intravenous iron infusions. Given the degree of the anemia and thrombocytopenia, extensive work-up was performed including flow cytometry, bone marrow biopsy, chest x-ray, and computed tomography (CT) of soft tissue neck. A CT abdomen/pelvis and a pelvic ultrasound (USG) were performed in view of her menorrhagia. USG venous duplex was performed for both lower limbs as she complained of bilateral aches and pains in both lower extremities during the hospitalization. CT angiography chest was performed to rule out pulmonary embolism. The peripheral blood smear showed marked thrombocytopenia without aggregation and hypochromic anemia with the presence of nucleated red blood cells and anisocytosis (Figure 1).

**Figure 2:**
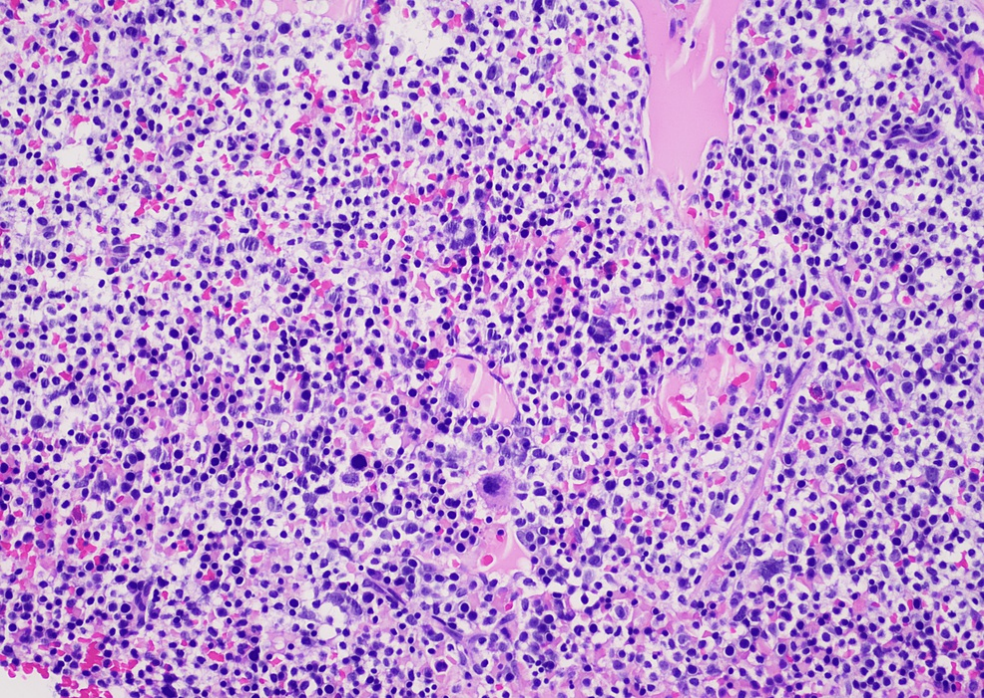
A hypercellular bone marrow packed with small leukemic cells having moderate light basophilic cytoplasms and circumferential hairy projections This image was originally published in the American Society of Hematology (ASH) Image Bank.

The bone marrow biopsy showed a hypercellular bone marrow with a 95% involvement by HCL. An adequate number of megakaryocytes were seen, and trace iron stores were noted. The reticulin stain demonstrated no significant increase in reticulin fibers (Figure 2).

**Figure 3:**
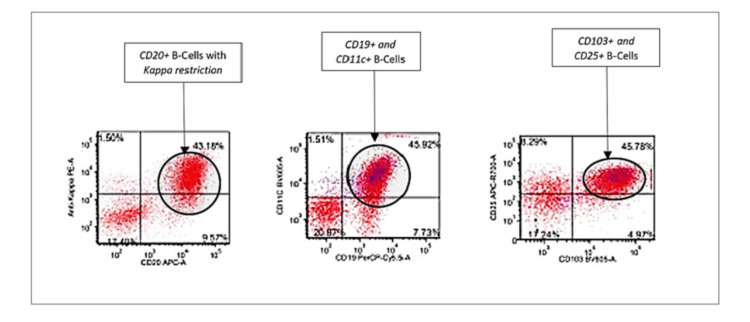
Flow cytometry showing clonal CD19 and CD20-positive B-cell population with kappa restriction and CD11c, CD25 and CD103 This image was originally published in the American Society of Hematology (ASH) Image Bank. CD: Cluster of differentiation.

**Figure 4:**
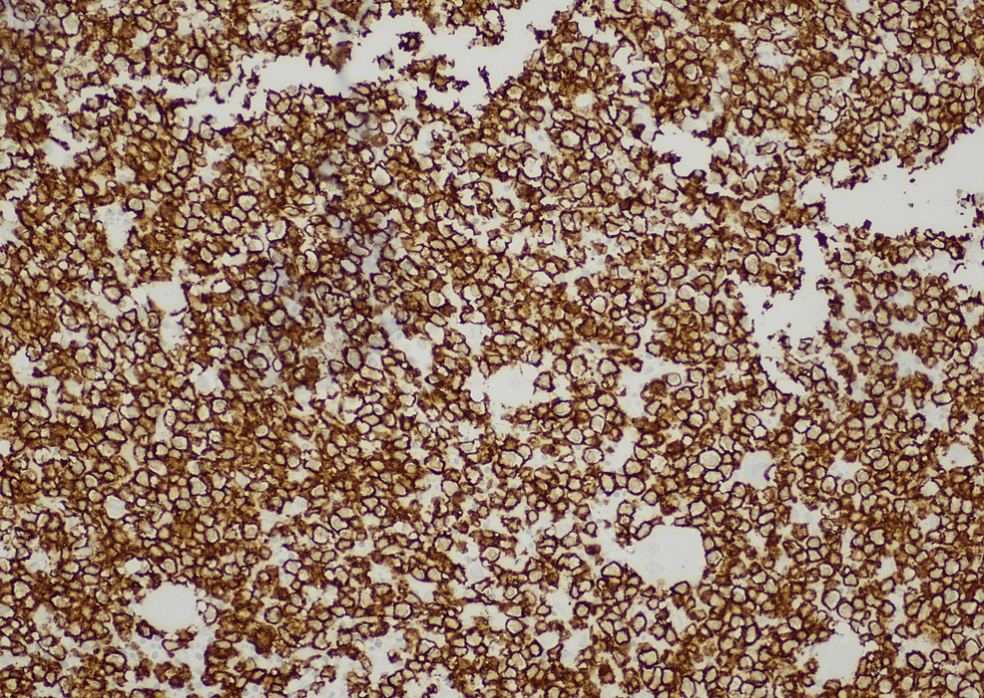
Bone marrow showing CD20-positive leukemic cells CD: Cluster of differentiation.

**Figure 5:**
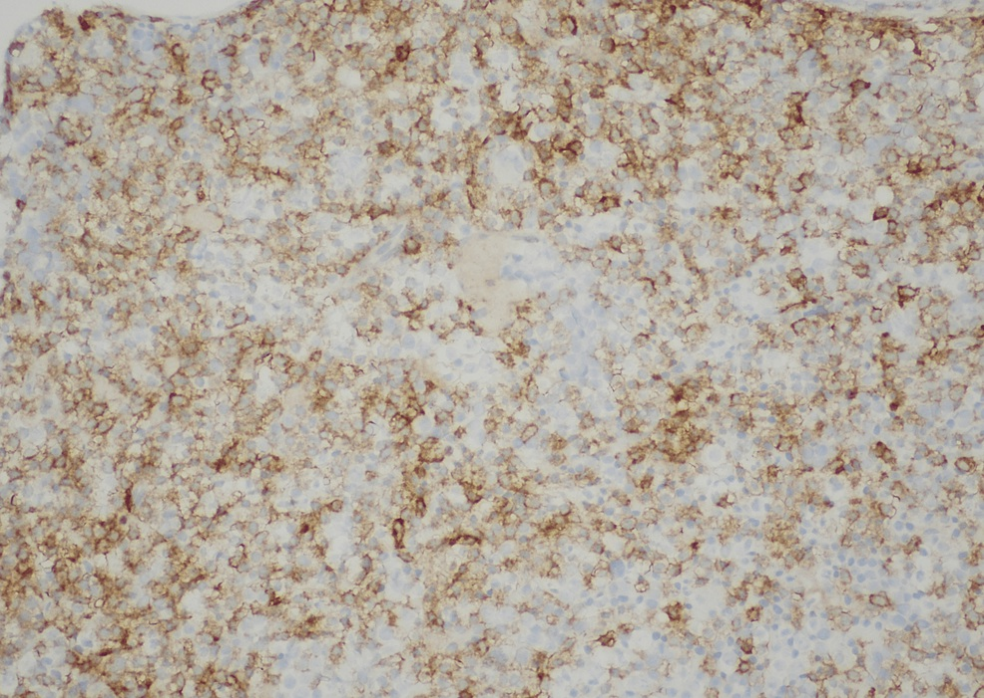
Bone marrow showing CD25-positive leukemic cells CD: Cluster of differentiation.

**Figure 6:**
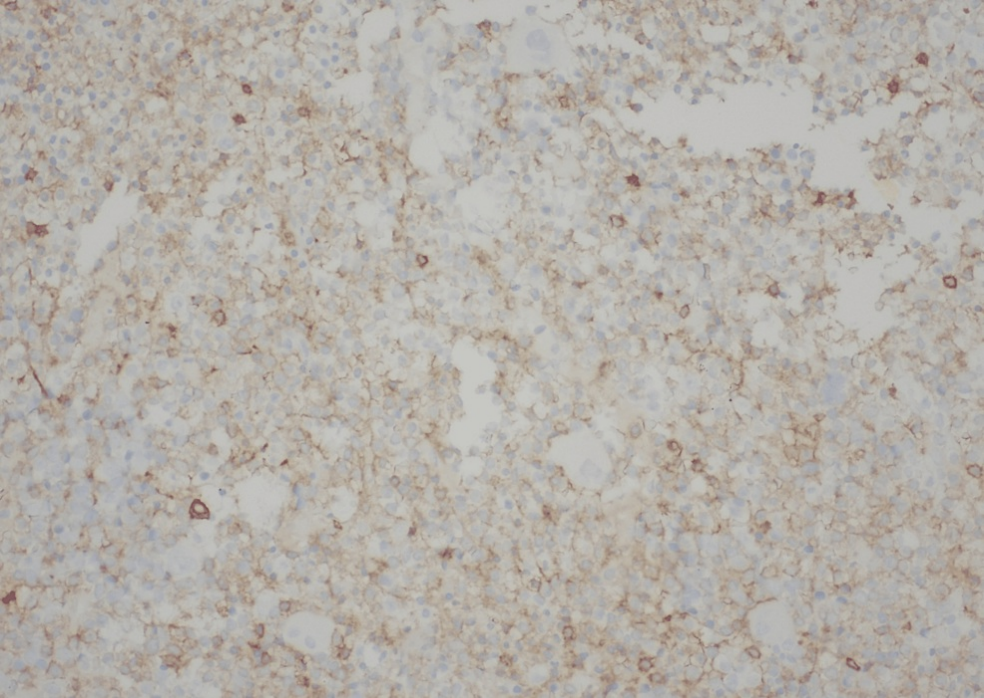
Bone marrow showing leukemic cells positively staining with Annexin A1

**Figure 7:**
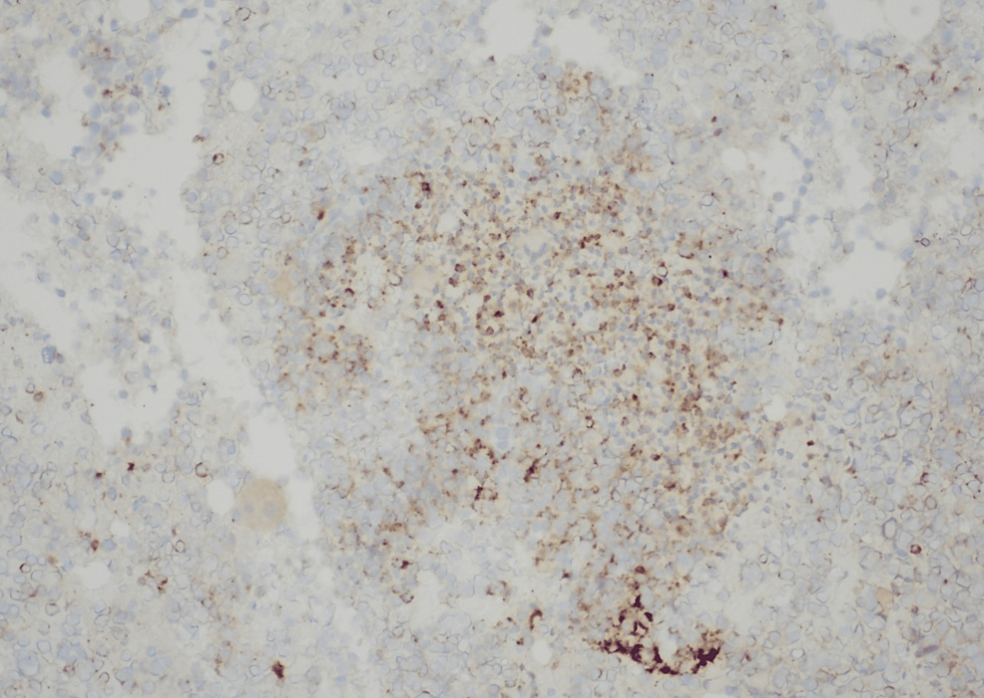
Bone marrow showing leukemic cells positively staining with TRAP stain TRAP: Tartrate-resistant acid phosphatase.

The patient's flow cytometry analysis revealed clonal cluster of differentiation 20 (CD20)-positive B cells with CD11c, CD25, CD103, CD10, and kappa (κ) light chain restrictions, which are highly suggestive of HCL (Figure 3). The leukemic cells were positive for CD20, CD25, CD10, Annexin A1, and tartrate-resistant acid phosphatase (TRAP) stain and negative for CD123 (Figures 4-7). The scattered T-cells were positive for CD3 and CD5. Next gene sequencing (NGS) was positive for BRAF Val600 Glu (B-RAF gene valine substituted for glutamate at position 600) mutation. Cytogenetic abnormality of 46,XX,t(12,22)(p13;q11.2){2}/46,XX{18} was also seen.

The CT soft tissue neck revealed mild lymphadenopathy of the right jugulodigastric region and left jugulodigastric region lymph nodes. CT abdomen/pelvis showed a large cyst in the left adnexa (3 cm x 4 cm), more likely to be of ovarian origin with mild ascites and a low-lying copper intra-uterine device (IUD). The patient’s further imaging revealed upper abdominal lymphadenopathy, mild retroperitoneal lymphadenopathy, and splenomegaly with a suggestion of a possible splenic infarct. The CT angiography showed no evidence of pulmonary embolism; the bilateral venous duplex USG was negative for deep vein thrombosis (DVT), and the chest x-ray was unremarkable. At the time of her discharge, her hemoglobin was 8.8 g/dL with a hematocrit of 28.3% and a platelet count of 56,000/µL. In regard to her menorrhagia and severe anemia, she was advised to consider the removal of the IUD and to regularly follow-up with her gynecologist.

Two weeks after her discharge, she presented to outpatient oncology for further follow-up. At her follow-up visit with oncology, she reported feeling much better since her discharge and denied any significant night sweats, fevers, or chills. The patient was informed of her diagnosis. However, she was unwilling to accept the significance of her leukemia and was reluctant to undergo any further treatment/intervention at this stage. This may be due to her lack of insurance or possible denial of the severity of her condition. Currently, treatment discussions are undergoing, and social workers and transitional care teams are being consulted to help the patient with the proper resources needed for the timely management of her condition.

## Discussion

Leukemia is characterized by a hairy cell, a clonal B-lymphocyte, which infiltrates the reticuloendothelial system leading to bone marrow failure, splenomegaly, and hepatomegaly [1-3]. The etiopathogenesis of HCL has strongly been associated with the BRAF-V600E kinase-activating mutation, which activates the rapidly accelerating fibrosarcoma-mitogen-activated protein kinase-extracellular signal-regulated kinase (RAF-MEK-ERK) pathway [2,4]. The continued activity of this signaling pathway leads to increased cellular proliferation and anti-apoptotic features of the cell, ultimately leading to malignancy [4]. The second most common mutation seen in HCL is that of cyclin-dependent kinase inhibitor 1B/p27 (CKDN1B/p27 cell cycle inhibitor inactivation) [5]. Studies have also reported cytogenetic abnormalities involving chromosomes 11, 12, 14, and 22 along with chromosome 5 trisomy to be associated with HCL [4,6].

Patients with HCL often present with nonspecific symptoms such as fatigue, weakness, abnormal bleeding, or symptoms of ongoing infection [1,3,5]. Although splenomegaly is a classic symptom, which is present in almost 90% of patients, it may be seen less frequently due to earlier detection of the disease [3]. More frequently, patients may present with the incidental finding of pancytopenia [1,3,5].

Definitive diagnosis of HCL is obtained by peripheral blood smear, immunophenotypic profile, and bone marrow biopsy with aspirate [3,5]. Peripheral blood smear review with a differential count in the case of HCL typically shows pancytopenia with normocytic-normochromic anemia, neutropenia, monocytopenia, and thrombocytopenia [1,3,5]. Monocytopenia is a relatively specific and sensitive manifestation of the classic variant HCL. Diagnostic hairy cell, a medium-sized cell with moderately abundant pale blue cytoplasm, and circumferential serrated “hair-like” cytoplasmic projections with a round-well defined nucleus can be seen on peripheral blood smear and also in the bone marrow [3,5]. Immunophenotyping/flow cytometry of peripheral blood mononuclear cells reveals light chain restriction of kappa (κ) or lambda (λ) expressing B-cell populations [3]. The characteristic flow cytometry markers for HCL are CD11c+, CD25+, CD103+, and CD123+ along with typical B-cell markers of CD19+, CD20+, or CD22+ [3,5]. The trephine bone marrow biopsy with aspirate is usually performed to confirm the diagnosis of HCL and to assess the extent of infiltration of the bone marrow. A “dry tap” or inability to attain a successful bone marrow aspirate is frequently encountered [2]. This indicates extensive bone marrow fibrosis. The extent of bone marrow leukemic cell infiltration can be evaluated via immunohistochemical stains for CD20, Annexin-A1, cyclin D1, and VEI/BRAFV600E [3,4]. Together, the peripheral blood smear findings, flow cytometry, and bone marrow biopsy confirm the diagnosis of HCL. The required imaging should also be performed to assess for internal lymphadenopathy and splenomegaly [1,3,5].

There is an increased risk of secondary malignancies associated with HCL such as melanomas, prostate and gastrointestinal malignancies, and non-Hodgkin’s lymphomas [7]. The median survival can range up to normal life expectancies with timely medical treatment [7].

The current treatment guidelines for HCL recommend primary nucleoside analog induction therapy with either cladribine or pentostatin in the absence of renal impairment or active infection, whereas patients who are asymptomatic do not require immediate therapy but need close clinical follow-up [3,5,8]. In general, the hematological parameters indicating a necessity for treatment include at least one of the following: hemoglobin level less than 11 g/dL, platelet count less than 100,000/µL, or absolute neutrophil count of less than 1000/µL or severe systemic symptoms [3,5,8].

Cladribine, due to its favorable toxicity profile, is regarded as the first-line chemotherapy agent (0.1 mg/kg/day via continuous intravenous infusion for seven days, or 0.14 mg/kg/day intravenously over two hours once per day for five days, or 0.1-0.14 mg/kg/day subcutaneously once per day for five days) [3,8]. Pentostatin (4 mg/kg/day subcutaneously once per day for five days) can also be given [3,8]. In cases uncontrolled with cladribine and pentostatin maintenance therapy, other agents such as rituximab, vemurafenib, and interferon-alpha (IFN-α) can be used [8]. Splenectomy may be considered a temporary measure in patients with symptomatic splenomegaly and severe pancytopenia due to recurrent splenic sequestrations. However, due to its high association with perioperative morbidity and mortality, it is rarely indicated in the current realm of supportive care [5,8].

## Conclusions

The reason why it is almost a near miss is that HCL can present with a vast array of symptoms, ranging from being completely asymptomatic to massive symptomatic splenomegaly and pancytopenia. It can present atypically, has an indolent course, and therefore can easily be overlooked initially. This unusual case report of a middle-aged woman with HCL reinforces the paramount importance of thorough evaluation and vigilant investigations in any patient presenting with pancytopenia for timely diagnosis and management. Socioeconomic barriers may prove to be further detrimental as seen in this case as they may further delay diagnosis and management.
